# Genotype–phenotype correlations in CPT1A deficiency detected by newborn screening in Pacific populations

**DOI:** 10.1002/jmd2.12271

**Published:** 2022-03-26

**Authors:** Isaac Bernhardt, Emma Glamuzina, Leah K. Dowsett, Dianne Webster, Detlef Knoll, Kevin Carpenter, Michael J. Bennett, Michelle Maeda, Callum Wilson

**Affiliations:** ^1^ National Metabolic Service Auckland City Hospital and Starship Children's Hospital Auckland New Zealand; ^2^ Department of Pediatrics University of Hawai'i John A. Burns School of Medicine Honolulu Hawai'i USA; ^3^ Hawai'i Community Genetics Honolulu Hawai'i USA; ^4^ Newborn Metabolic Screening Unit Auckland City Hospital Auckland New Zealand; ^5^ Chemical Pathology (Section New Born Screening) Auckland City Hospital Auckland New Zealand; ^6^ Human Genetics Society of Australasia Sydney Australia; ^7^ Perelman School of Medicine University of Pennsylvania Philadelphia Pennsylvania USA; ^8^ State of Hawai'i Department of Health Children with Special Health Needs Program Honolulu Hawai'i USA

**Keywords:** carnitine palmitoyltransferase type 1 deficiency, CPT1A, fatty acid oxidation disorders, newborn screening

## Abstract

Carnitine palmitoyltransferase 1A (CPT1A) deficiency is a long chain fatty acid oxidation disorder, typically presenting with hypoketotic hypoglycaemia and liver dysfunction during fasting and intercurrent illness. Classical CPT1A deficiency is a rare disease, although a milder ‘Arctic variant' (p.P479L) is common in the Inuit population. Since the introduction of expanded metabolic screening (EMS), the newborn screening programmes of Hawai'i and New Zealand (NZ) have detected a significant increase in the incidence of CPT1A deficiency. We report 22 individuals of Micronesian descent (12 in NZ and 10 in Hawai'i), homozygous for a *CPT1A* c.100T>C (p.S34P) variant detected by EMS or ascertained following diagnosis of a family member. No individuals with the Micronesian variant presented clinically with metabolic decompensation prior to diagnosis or during follow‐up. Three asymptomatic homozygous adults were detected following the diagnosis of their children by EMS. CPT1A activity in cultured skin fibroblasts showed residual enzyme activity of 26% of normal controls. Secondly, we report three individuals from two unrelated Niuean families who presented clinically with symptoms of classic CPT1A deficiency, prior to the introduction of EMS. All were found to be homozygous for a *CPT1A* c.2122A>C (p.S708R) variant. CPT1A activity in fibroblasts of all three individuals was severely reduced at 4% of normal controls. Migration pressure, in part due to climate change may lead to increased frequency of presentation of Pacific peoples to regional metabolic services around the world. Knowledge of genotype–phenotype correlations in these populations will therefore inform counselling and treatment of those detected by newborn screening.

**Synopsis**

Novel *CPT1A* variants cause classical CPT1A deficiency in the Niuean population (c.2122A>C) and apparently benign CPT1A deficiency in the Micronesian population (c.100T>C).

## 1 INTRODUCTION

Carnitine palmitoyltransferase I (CPT1) catalyses the synthesis of long chain acylcarnitines from their fatty acyl‐CoA derivatives. These are then translocated into the mitochondrial matrix, converted back to their acyl‐CoA origins and undergo ß‐oxidation to produce acetyl‐CoA and ketone bodies. There are three distinct genetic isoforms: CPT1A is expressed primarily in the liver, CPT1B in heart and skeletal muscle, and CPT1C in the brain.^1^

CPT1A deficiency (OMIM#255120) is a long chain fatty acid oxidation disorder (FAOD) with a predominantly hepatic phenotype. The classical presentation is with hypoketotic hypoglycaemia and hepatitis (with or without hyperammonaemia) provoked by fasting, usually in the context of intercurrent illness. The other classic FAOD symptoms of rhabdomyolysis and cardiomyopathy are not usually a feature, due to the normal function of CPT1 isoforms in heart and skeletal muscle.^1^, ^2^

Expanded metabolic screening (EMS) using tandem mass spectrometry (TMS) was introduced in Hawai'i in 2003 and New Zealand (NZ) in 2006. EMS can identify patients with CPT1A deficiency by their high free carnitine (C0) levels and high C0/(C16 +  C18) ratios.^1^, ^2^ The disease appears to be rare with few symptomatic patients reported. There is a well described *CPT1A* variant that is very common in the Inuit population. There is some evidence that it is associated with a greater incidence of early childhood death when present in the homozygous state.^2^, ^3^, ^4^

The newborn screening services of NZ and Hawai'i each screen large numbers of newborns of Pacific ancestry. Pacific refers to people from the geographically, ethnically and culturally diverse regions of Polynesia (including NZ, Samoa, Tonga, Niue, Hawai'i and the Cook Islands), Micronesia (Kiribati, Nauru, Caroline and Marshall Islands) and Melanesia (Vanuatu, New Caledonia, Fiji and Solomon Islands). These two screening units, and the clinical services associated with them, have identified two distinct groups of children with CPT1A deficiency from Niue and Micronesia. This paper describes the experience of EMS for CPT1A deficiency in Hawai'i and NZ, and delineates the phenotype associated with two previously unreported variants.

## 2 METHOD

This was a retrospective review of all cases diagnosed with CPT1A deficiency by the metabolic services and newborn screening units of Hawai'i and NZ, identified by audit of respective databases. For all cases identified, clinical data from medical records were interrogated for evidence of symptomatic CPT1A deficiency, including encephalopathy, hypoketotic hypoglycaemia and liver dysfunction, particularly during fasting or episodes of increased metabolic demand, including illnesses and surgery.

EMS (where performed) was by TMS on dried blood spots (DBS) collected at 48–72 h after birth. C0/(C16 +  C18) ratio was used as the primary screening marker in Hawai'i with a cut‐off of 100. In NZ, the combination of raised C0 (cut‐off 60 μmol/L) and C0/(C16 +  C18) ratio (cut‐off 70) was used.

CPT1 enzyme activity (where performed) was measured in cultured skin fibroblasts, by published methods.^5^ CPT1 activity was estimated as the malonyl‐CoA sensitive CPT component, or by oxidation of (9,10‐^3^H) myristate, (9,10‐^3^H) palmitate, and (9,10‐^3^H) oleate.

Molecular analysis by Sanger sequencing of the *CPT1A* gene was performed at clinical molecular genetics laboratories including Children's Hospital of Philadelphia, Emory Genetics Laboratory, Invitae and Labplus (Auckland, NZ).

## 3 RESULTS

Twenty‐five individuals with CPT1A deficiency were identified. Of these, three were homozygous for the *CPT1A* c.2122A>C (p.S708R) variant, and the remaining 22 were homozygous for *CPT1A* c.100T>C (p.S34P). All individuals had clinical review and genetic counselling following diagnosis, provided by metabolic services in Hawai'i and NZ.

### 3.1 Micronesian variant

Twenty‐two individuals were found to be homozygous for the *CPT1A* c.100T>C (p.S34P) variant. Twelve were identified in NZ, all of whom were of Kiribati ethnicity. Seven were diagnosed by EMS and five following the diagnosis of an affected family member; none presented clinically. Avoidance of prolonged fasting was recommended and a written emergency regimen for sick‐day management was provided by the treating physician at the time of clinical review, for all cases except for two asymptomatic adults. One individual required intensive care admission in infancy due to sepsis with a retropharyngeal abscess, but remained stable from a metabolic perspective, with only a mildly elevated creatine kinase. Another developed mild acidosis during severe gastroenteritis with profuse watery diarrhoea, treated with intravenous fluid therapy. All other cases have remained asymptomatic (age range 5 weeks–33 years). Enzymology in cultured skin fibroblasts in an asymptomatic adult, showed residual CPT1 activity at 26% of normal controls, despite abnormal acylcarnitine metabolites consistent with CPT1A deficiency (Table 1).

**TABLE 1:** Micronesian cases (homozygous c.100T>C p.Ser34Pro)

Case	Ethnicity	Presentation	C0 (<60 μmol/L)	C0/C16 + C18^a^	CPT1 activity	Notes	Age at last follow‐up
New Zealand							
1	Kiribati	Diagnosed by EMS	248	147	N/A		6 years
2	Kiribati	Older sibling of case 1, no prior EMS	196	158	N/A		8 years
3	Kiribati	Diagnosed by EMS, younger sibling of case 1	167	84	N/A		5 years
4	Kiribati	Younger sibling of case 1, normal EMS on 2 occasions	Normal	Normal	N/A		2 years
5	Kiribati	Asymptomatic parent of case 1, no prior EMS	230	102	26%^b^		30 years
6	Kiribati	Asymptomatic parent of case 1, no prior EMS	Normal	Normal	N/A		29 years
7	Kiribati	Diagnosed by EMS	201	102.71	N/A	Episode of severe gastroenteritis in infancy with profuse watery diarrhoea, mild acidosis but normal glucose, treated with intravenous dextrose. Mild speech delay.	6 years
8	Kiribati	Asymptomatic parent of case 7, no prior EMS	Normal	Normal	N/A		33 years
9	Kiribati	Diagnosed by EMS, younger sibling of case 7	126	137	N/A	Hypoxic ischaemic encephalopathy (grade 2) secondary to cord prolapse at term. No developmental concerns at 3 years of age.	3 years
10	Kiribati	Diagnosed by EMS	166.8	115.53	N/A	ICU admission with bronchiolitis and large retropharyngeal abscess at 4 months with CK 728 μ/L, no other signs of metabolic decompensation	6 years
11	Kiribati	Diagnosed by EMS	165.6	127.11	N/A		1 year
12	Kiribati	Diagnosed by EMS	146	76	N/A		5 weeks
Hawai'i							
13	Marshall Islands	Diagnosed by EMS	N/R	295	N/A		12 years
14	Marshall Islands	Diagnosed by EMS	N/R	112	N/A		7 years
15	Marshall Islands	Diagnosed by EMS	N/R	140	N/A		4 years
16	Marshall Islands	Diagnosed by EMS	N/R	128.4	N/A		3 years
17	Marshall Islands	Diagnosed by EMS	N/R	147	N/A		3 years
18	Marshall Islands	Diagnosed by EMS	N/R	246.4	N/A	Cleft lip, tolerated surgery without additional metabolic intervention	2 years
19	Chuuk	Diagnosed by EMS	N/R	202	N/A	Elevated transaminases (ALT and AST) associated with viral illness, resolved without treatment	10 years
20	Chuuk	Diagnosed by EMS	N/R	153	N/A		9 years
21	Chuuk	Diagnosed by EMS	N/R	163.4	N/A		5 years
22	Pohnpei	Diagnosed by EMS	N/R	101	N/A		7 years

Abbreviations: ALT, alanine transaminase; AST, aspartate transaminase; C0, free carnitine; CPT, carnitine palmitoyltransferase; CK, creatine kinase; EMS, expanded metabolic screening; ICU, intensive care unit; N/A, not available; N/R, not recorded.

^a^ C0/C16 + C18 cut‐off <70 in New Zealand and <100 in Hawai'i.

^b^ CPT1 activity = 26% of normal controls (0.33 nmol/min/mg protein estimated as the malonyl‐CoA sensitive CPT component).

The EMS program of Hawai'i identified 10 individuals with raised C0/(C16 +  C18) (classified as screen positive). Subsequent acylcarnitine profiles for all patients, when performed, were normal. Sequencing of the *CPT1A* gene found all patients to be homozygous for *CPT1A* c.100C>T (p.S34P). Pedigree analysis identified all individuals to be of Micronesian descent, with the affected patients coming from the Marshall Islands (6), Chuuk (3), and Pohnpei (1). Specific dietary management was not instituted for these patients, and no episodes of metabolic decompensation were documented. None were hospitalised with metabolic crises in their lifetimes to date (age range 2–12 years). One patient had mildly elevated transaminases during a viral illness that resolved without treatment (Table 1).

### 3.2 Niuean variant

Three individuals homozygous for *CPT1A* c.2122A>C (p.S708R) were identified, from two unrelated families (Table 2). All were of Niuean ethnicity, and were born prior to the introduction of EMS. Two presented clinically with classical CPT1A deficiency symptoms, the third was an asymptomatic younger sibling, diagnosed prospectively. Significantly elevated creatine kinase in the setting of acute encephalopathy was noted in one individual, and chronic mild hyperammonaemia in adulthood in another. CPT1A activity in cultured skin fibroblasts was consistent with severe CPT1A deficiency in all three cases (Table 2).

**TABLE 2:** Niuean Cases (homozygous c.2122A>C; p.Ser708Arg)

Case	Presentation	Laboratory values at presentation	Additional investigations at presentation	Acylcarnitine profile^a^	Enzymology results (cultured skin fibroblasts)
1	Recurrent hypo‐ketotic hypoglycaemia in childhood. Encephalopathy at 25 years of age with mild hyper‐ammonaemia	Glucose = 5.8 mmol/L (3.5–5.4) CK = 82 U/L (30–180) NH_3_ = 75 μmol/L (<70) Urine organic acids normal	CT brain: mild cerebral atrophy, otherwise normal	C0 = 91 μmol/L (13–56) C2 = 5 μmol/L (3–23) Total carnitine = 98 μmol/L (21–70)	CPT1A activity = 4% (0.10 nmol/min/mg protein estimated as the malonyl‐CoA sensitive CPT component)
2	Encephalopathy at 7 months of age, with seizures, hypoketotic hypoglycaemia, liver dysfunction, coagulopathy, acute kidney injury, and global cerebral injury	Glucose = 2.7 mmol/L (3.5–5.4) B‐(OH)butyrate = 0.6 mmol/L (0–0.3) Ca = 1.44 mmol/L (2.1–2.55) TG = 40 mmol/L (0.5–2.3) CK = 4707 U/L (30–180) NH_3_ normal Urine organic acids normal	Liver biopsy: severe and diffuse micro‐ and macro‐vascular steatosis Bone marrow: lipid inclusions Echocardiogram: normal	N/A	Markedly reduced oxidation of C14 & C16 Normal C4 dehydrogenase and C16 dehydrogenase activity Ratio C4/C16 activity normal
3	Sibling of affected family member (case 2), asymptomatic at diagnosis at 14 months of age, subsequent recurrent episodes of mild metabolic decompensation.	Nil.	Nil.	N/A	Oxidation of C18:1 & C14 = 4% of normal controls Ratio C18:1/C16 activity = 0.9 (normal)

Abbreviations: Ca, calcium; CK, creatine kinase; CPT, carnitine palmitoyltransferase; CT, computer tomography; N/A, not available; NH_3_, ammonia; TG, triglycerides.

^a^ Incomplete acylcarnitine profile results in these cases reflects historical limitation in availability.

## 4 DISCUSSION

CPT1A deficiency is a rare disease. Excluding the common ‘Arctic variant' (p.P479L), there are fewer than 60 affected individuals reported in the literature.^2^ The severe phenotype of hypoketotic hypoglycaemia with acute hepatic failure associated with CPT1A deficiency is well described.^2^ We report three individuals from two families, homozygous for a previously unreported Niuean variant (p.S708R) who presented clinically with a classical, severe phenotype. CPT1A enzyme activity was correspondingly reduced at 4% of normal controls. Of interest, one patient had moderately raised creatine kinase, and while this has previously been reported in CPT1A deficiency,^6^ the pathogenesis is not well understood, given the presumed normal activity of the muscle isoform CPT1B. Similarly, hyperammonaemia has been reported in CPT1A deficiency and in other long chain FAODs,^1^, ^2^ although the mild persistence of this abnormality even in the well state was notable. Following the introduction of EMS in NZ and Hawai'i, no further cases of Niuean‐variant CPT1A deficiency have been detected.

By contrast, the Micronesian variant (p.S34P) is associated with residual enzyme activity at 26% of normal controls. While most individuals had abnormal acylcarnitine metabolites on EMS, one had normal biochemistry at the time of newborn screening (Table 2), therefore it is likely that other individuals homozygous for this variant escape detection by EMS. Three cases were asymptomatic adults, diagnosed after the detection of affected offspring, and of these only one had abnormal acylcarnitine metabolites. There have been no episodes of significant metabolic decompensation in any of the 22 cases, including 10 patients who were not treated with dietary management, and one who was admitted to the intensive care unit with sepsis. Therefore, the risk of clinical sequelae associated with the Micronesian variant is likely to be significantly lower than with classical CPT1A deficiency.

The *CPT1A* ‘Arctic variant' (p.P479L) is associated with comparable residual enzymatic activity at 20% of normal controls, and impaired fasting ketogenesis has been demonstrated in homozygous infants.^7^ While hypoketotic hypoglycaemia has not been documented in individuals homozygous for the Micronesian variant (p.S34P), a similar mild impairment in ketogenesis cannot be ruled out in the absence of formal fasting studies. However, it seems likely that the vast majority of these individuals would previously have remained undiagnosed.

Prior to the introduction of EMS, CPT1A deficiency was an extremely rare disease in NZ and Hawai'i. Following the establishment of Collaborative Laboratory Integrative Reports in 2004,^8^ only 27 cases of CPT1A deficiency have been detected by participating newborn screening programmes. NZ is significantly over‐represented with 11 cases, although as outlined here these cases are probably benign. To our knowledge, no individuals with the Micronesian variant have presented clinically with symptoms of CPT1A deficiency, prior to or following the introduction of EMS.

The carrier frequency of the Micronesian variant (p.S34P) has not been determined, but it is likely to be common in the Micronesian population, based on the unexpectedly high number of homozygotes detected since the introduction of EMS. The calculated incidence in the screened population is 1/128 500 in NZ and 1/28 500 in Hawai'i, however the true incidence of Micronesian variant CPT1A deficiency is likely somewhat higher, as suggested by the finding of normal EMS in one individual.

The ‘Arctic variant' (p.P479L) is highly prevalent in the Inuit population, with a carrier frequency of 0.68–0.85.^4^ This suggests a survival advantage in heterozygous carriers, further evidenced by the highly conserved adjacent genomic region, the largest selective sweep described in humans.^9^, ^10^ The Arctic variant may confer a selective advantage in carriers due to metabolic adaptation to the traditional Inuit diet.^10^ Due to a poor soil and a relative lack of fresh water compared to other Pacific islands, the traditional Kiribati diet was highly dependent on seafood and coconuts, and thus high in protein and fat but very low in carbohydrates.^11^ This is very similar to the Inuit diet and suggests there may have been a similar evolutionary selective advantage in carriers of the Micronesian variant.^10^

To our knowledge, this is the first published report of both the Niuean (p.S708R) and Micronesian (p.S34P) variants. These variants were both reported as likely pathogenic by clinical genomics laboratories. Both variants result in a non‐conservative amino acid substitution at a position which is highly conserved between species. The Micronesian (p.S34P) variant is located in the N‐terminal of the protein (residues 1–47),^12^ and of five other missense variants in this region reported to the ClinVar database, none have been reported as pathogenic or likely pathogenic.^13^ By contrast, the Niuean variant (p.S708R) is located in the catalytic domain of the protein, and adjacent pathogenic missense variants (p.G709E) and (p.G710E) have been shown to abolish CPT1A catalytic activity, as both are located in the hydrophobic core of the catalytic site.^12^

While these variants are absent in population genomic databases, minority populations including the indigenous Pacific populations, are under‐represented in these databases.^14^ Therefore, interpretation of newborn screening and genomic results in these populations is challenging. Identification of benign variants by newborn screening can result in unnecessary treatment, with psychosocial impacts on the individual, family and wider community.^15^ Recognition of the probably benign phenotype associated with the Micronesian variant should reduce the burdens associated with over‐treatment, including impacts on healthcare systems. Accordingly, we suggest a protocol whereby the Micronesian variant is confirmed by urgent *CPT1A* sequencing performed on the DBS sample, in screening programmes with significant Pacific populations. This approach, previously described for a benign Citrullinaemia type 1 variant,^16^ allows rapid identification of individuals who are unlikely to benefit from disclosure of benign variants detected by EMS.

The introduction of EMS in Hawai'i and NZ has increased the detection of CPT1A deficiency, and our experience is consistent with a strong genotype–phenotype correlation in individuals homozygous for previously unreported Niuean and Micronesian *CPT1A* variants. While Niue and the countries comprising Micronesia do not have their own screening programmes, individuals with these variants are likely to be detected by other regional newborn screening programmes, especially in North America and Australia. Climate change is expected to place severe migration pressure on many island nations in the Pacific and elsewhere due to sea‐level rise, and an increase in migration is predicted.^17^ Of these, Kiribati is particularly vulnerable, with an average altitude of just 1.8 m.^17^ Accordingly, knowledge of the genetic variome and genotype–phenotype correlations in these populations is vital to guide counselling and clinical management of those detected by newborn screening.

## CONFLICT OF INTEREST

Isaac Bernhardt, Emma Glamuzina, Leah Dowsett, Dianne Webster, Detlef Knoll, Kevin Carpenter, Michael Bennett, Michelle Maeda and Callum Wilson declare that they have no conflict of interest.

## ETHICS STATEMENT

All procedures followed were in accordance with the ethical standards of the responsible committee on human experimentation (institutional and national) and with the Helsinki Declaration of 1975, as revised in 2000 (5). No identifiable information is included in this article.

## Data Availability

My manuscript has no associated data.
